# Vascular thromboses with retroperitoneal fibrosis: a case report

**DOI:** 10.1186/s13256-021-03235-0

**Published:** 2022-01-26

**Authors:** Hanane Charaf, Rachida Zahraoui, Mouna Soualhi, Nezha Rguig, Jamal Eddine Bourkadi, Daoud Ali Mohamed, Nasser Ittimad

**Affiliations:** 1 Pneumo-Phtisiology Department, Faculty of Medicine and Pharmacy, Mohamed V University, Moulay Youssef Hospital, Rabat, Morocco; 2 Radiology Department, Faculty of Medicine and Pharmacy, Mohamed V University, Ibn Sina Hospital, Rabat, Morocco

**Keywords:** Granulomatosis with polyangiitis, Retroperitoneal fibrosis, Venous thromboembolism, Cytoplasmic antineutrophilic antibodies

## Abstract

**Background:**

Granulomatosis with polyangiitis is a systemic inflammatory disease characterized by necrotizing vasculitis that affects small- and medium-sized blood vessels. Granulomatous inflammation affects the lungs, ears, nose, and throat, and commonly affects the kidneys, although the retroperitoneal tissue is rarely affected. Several studies have reported an increased risk of venous thromboembolism. Early diagnosis and treatment are of vital importance due to the rapid progression of the disease.

**Case presentation:**

We present the case of a 66-year-old Moroccan man followed for bilateral jugular thrombosis. Cavitary pulmonary nodules and retroperitoneal fibrosis with thrombosis involving several vascular territories were detected on thoracoabdominopelvic computerized tomography scan. Laboratory analyses revealed that the patient was positive for cytoplasmic antineutrophilic antibodies. The diagnosis of granulomatosis with polyangiitis was retained. Treatment with glucocorticoids and immunosuppressive agents resulted in significant clinical and radiological improvement over the following months.

**Conclusions:**

We describe the diagnostic steps and the difficulty of managing this patient. Rare manifestations, such as retroperitoneal fibrosis, have been reported in the literature in association with granulomatosis with polyangiitis, and should not delay the diagnosis and treatment of granulomatosis with polyangiitis owing to its severity.

## Introduction

Granulomatosis with polyangiitis (GPA) is a necrotizing vasculitis affecting predominantly small to medium vessels (capillaries, venules, arterioles, arteries, and veins) [1]. This condition was previously known as Wegener’s granulomatosis. It is a rare disease, with an incidence in Europe of 2.1–14.4 cases/million, and it occurs in all age groups [2]. An increased incidence of various vascular events was demonstrated among GPA patients. We have shown that GPA patients are at increased risk of hospitalization for ischemic heart disease manifestations compared with age- and sex-matched controls [3]. For venous thromboembolic events (VTE), Merkel *et al.* reported a high occurrence of pulmonary embolism (PE) and deep venous thrombosis (DVT) among GPA patients included in a randomized therapeutic trial [4]. Among the observed VTEs, 81% occurred in patients with active or recently active vasculitis. Alvise Berti *et al.* found that, for patients with cytoplasmic antineutrophilic antibodies (ANCA)-associated vasculitis (AAV), the hazard ratio (HR) for venous thromboembolism VTE was 3.26 [95% confidence interval (CI) 0.84–12.60] and significantly increased for peripheral vascular disease DVT (HR 6.25, 95% CI 1.16–33.60), but not for pulmonary embolism (PE; HR 1.33, 95% CI 0.23–7.54) [5]. Other structures such as the retroperitoneal tissue, or large vessels including the aorta, are rarely involved. Retroperitoneal fibrosis incidence is 0.1 per 100,000 population/year with a prevalence of 1.4 per 100,000 population. Seventy percent of cases are idiopathic [2].

We present a case of multiple vascular thromboses, revealing granulomatous polyangiitis associated with retroperitoneal fibrosis without periaortitis. The association of retroperitoneal fibrosis with c-ANCA or p-ANCA positive systemic vasculitis is described in the literature, not only for granulomatosis with polyangiitis but also in other vasculitides such as Churg–Strauss syndrome [2].

The diagnosis of this association is lengthy and costly, with a lot of exploration [positron emission tomography (PET) scan and anatomopathological analysis of biopsies], which causes a delay in diagnosis that can expose the patient to complication risk [2, 3, 5].

Our observation describes the diagnostic particularity and management of this association, and we insist that retroperitoneal fibrosis should not delay the diagnosis of GPA or defer its therapeutic management.

## Case description

**Fig. 1:**
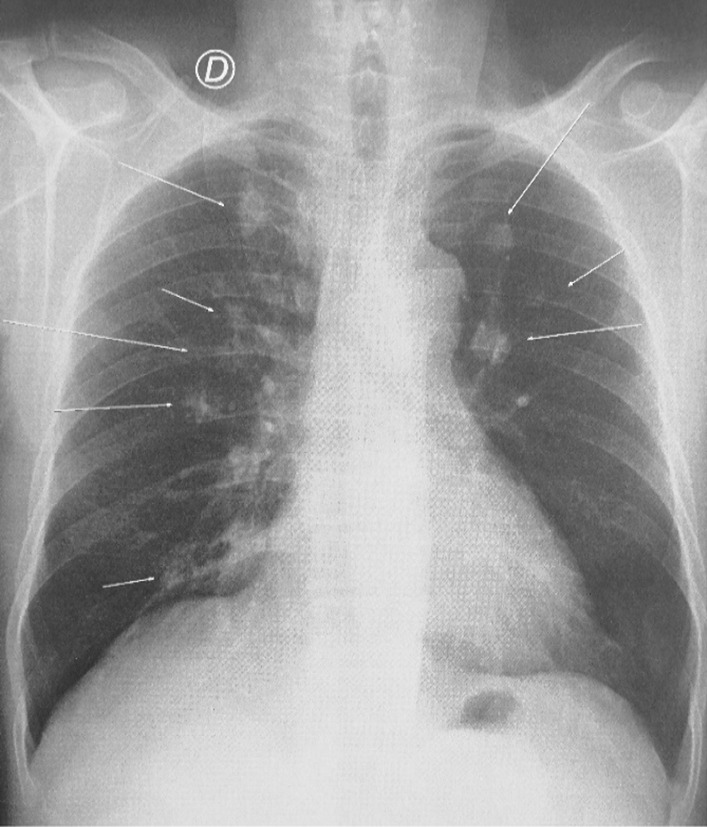
The frontal chest X-ray shows several excavated opacities

**Fig. 2:**
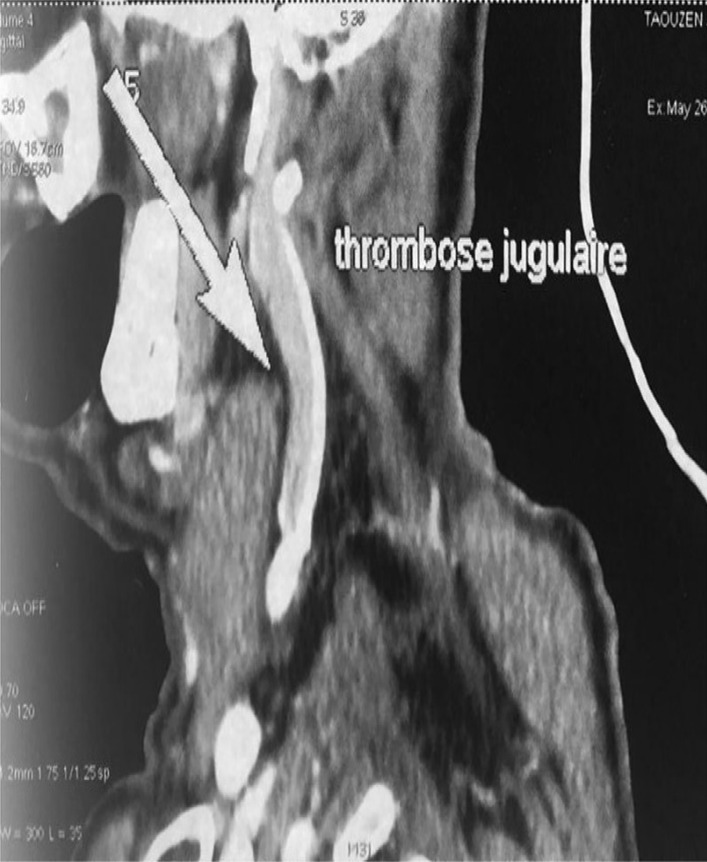
Partial thrombosis of the jugular vein

**Fig. 3:**
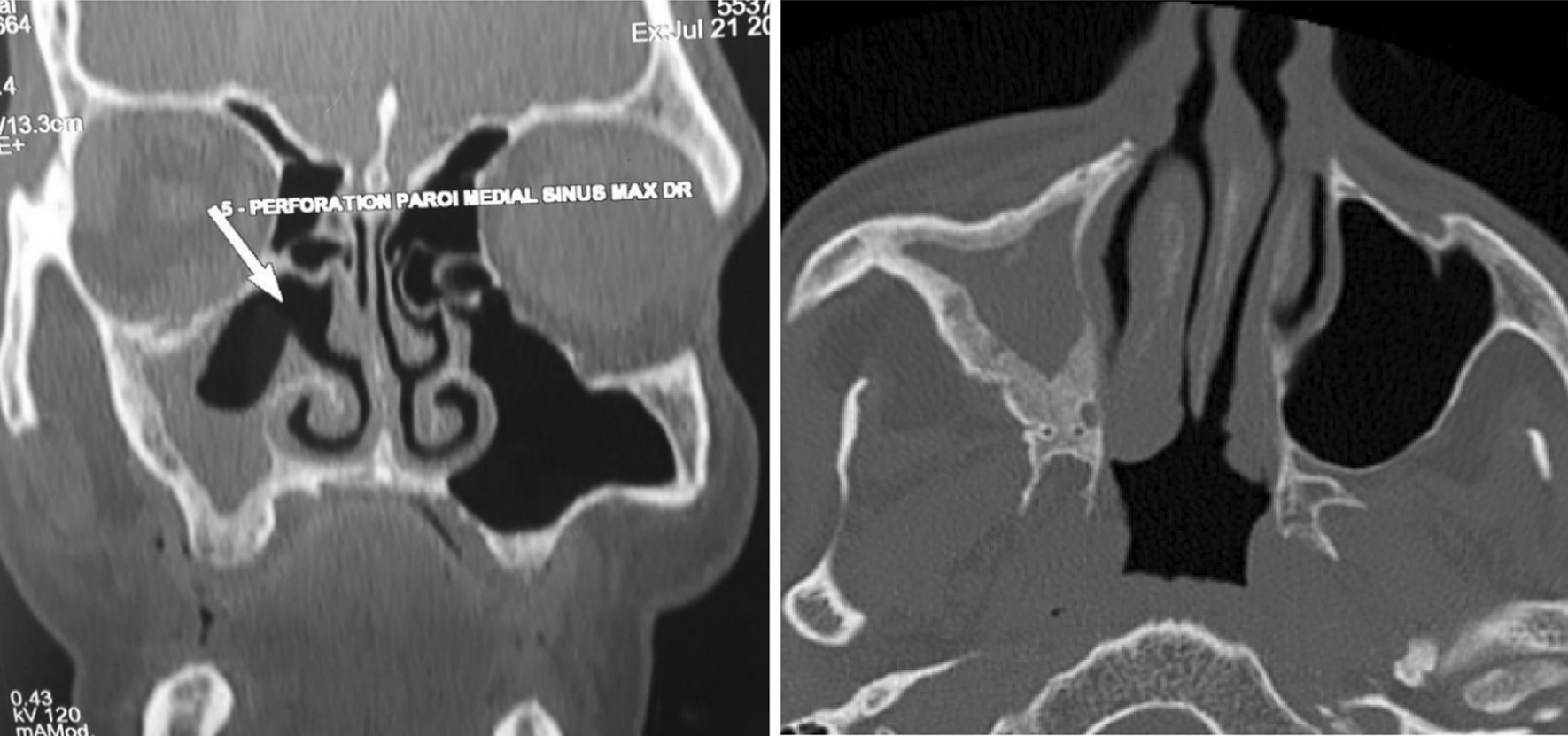
Sinus CT scan, axial section, frontal section: filling of the right maxillary sinus (sinusitis) with an erosion of the bone wall

**Fig. 4:**
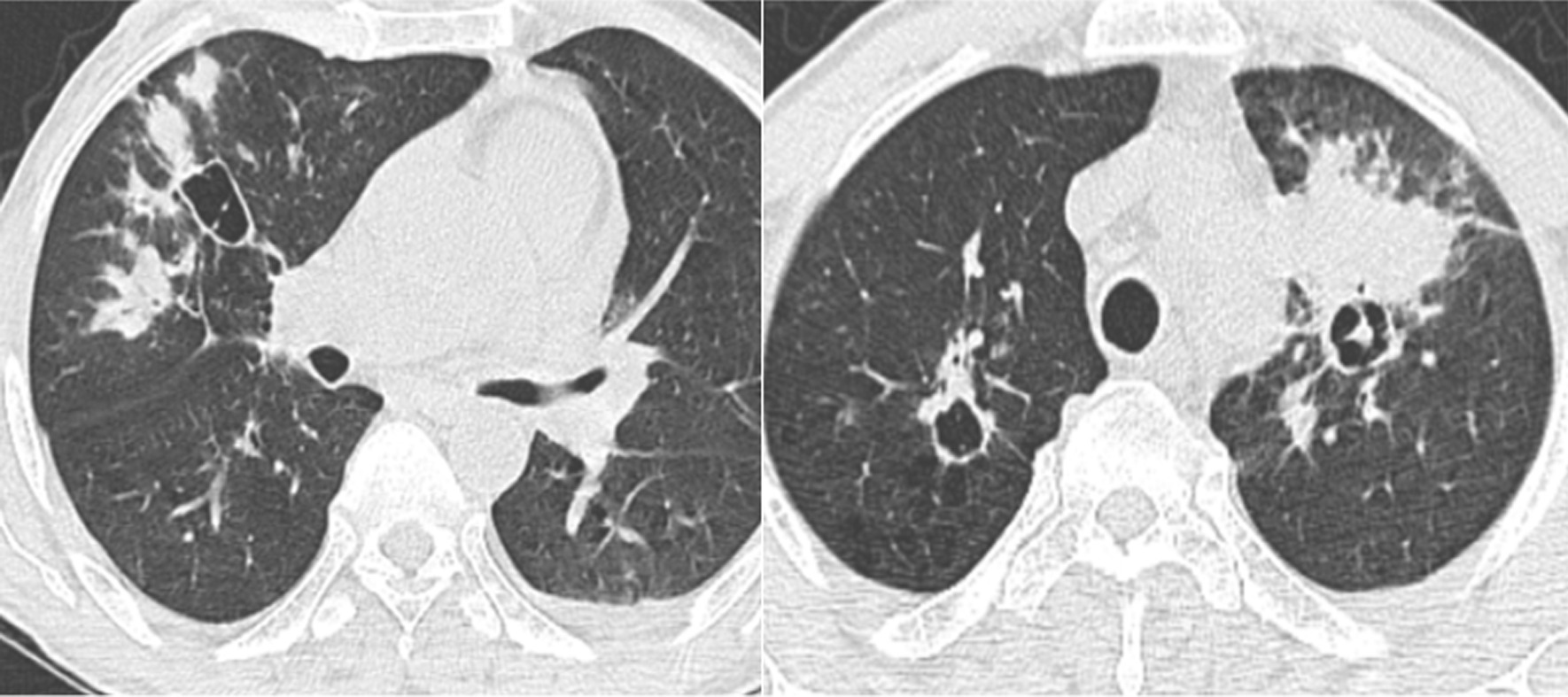
Thoracic CT scan, parenchymal window, axial sections: multiple excavated intraparenchymal lung nodules, irregularly contoured, thick-walled

**Fig. 5:**
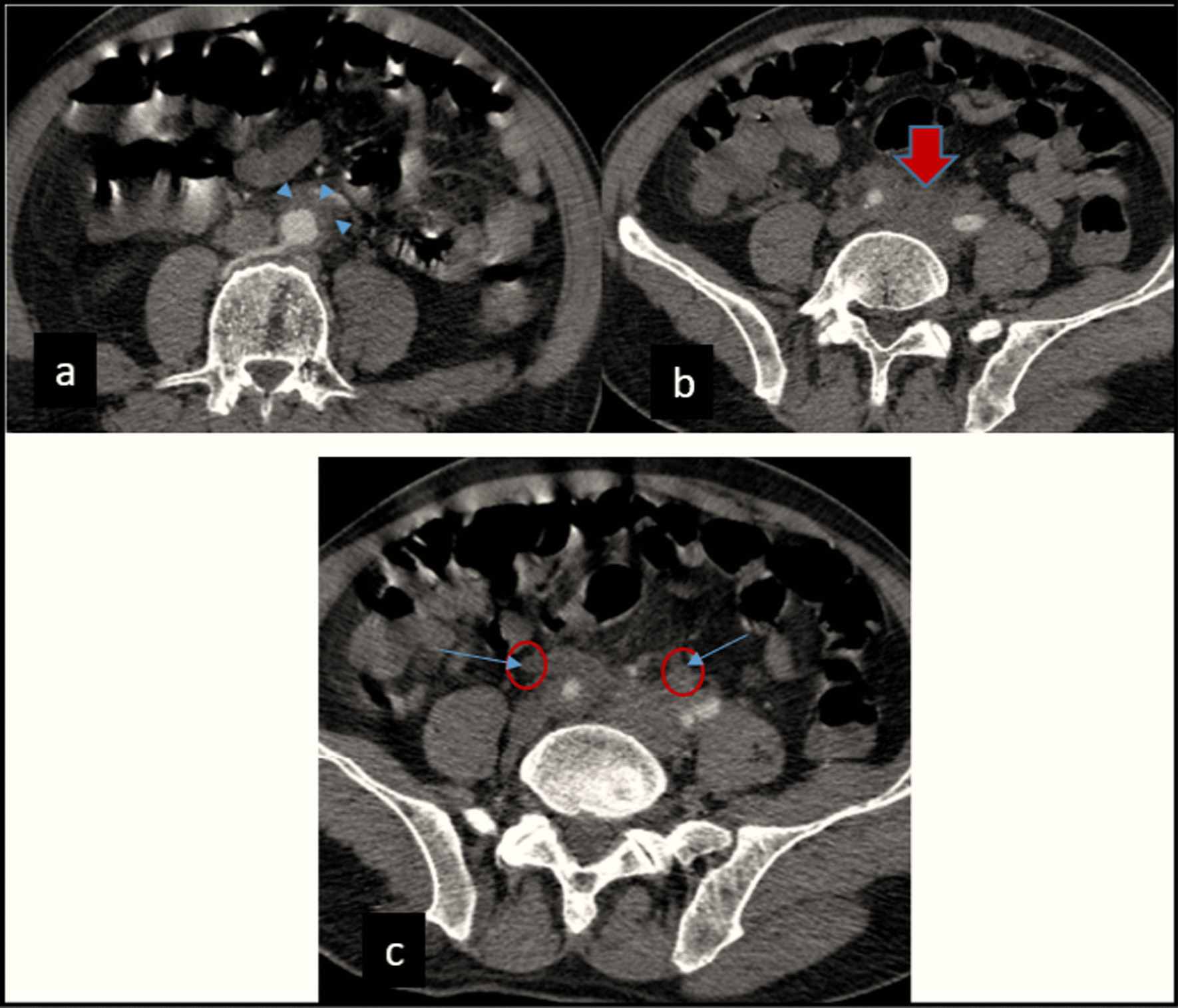
Abdominal CT scan, axial section:; tissue density infiltrate (or sleeve), homogeneous, perivascular, sheathing the abdominal aorta (**a**, arrowheads), extending to the iliac arteries (**b**, red arrow) and iliac ureters (**c**, blue arrows) in favor of retroperitoneal fibrosis (RFP)

**Fig. 6:**
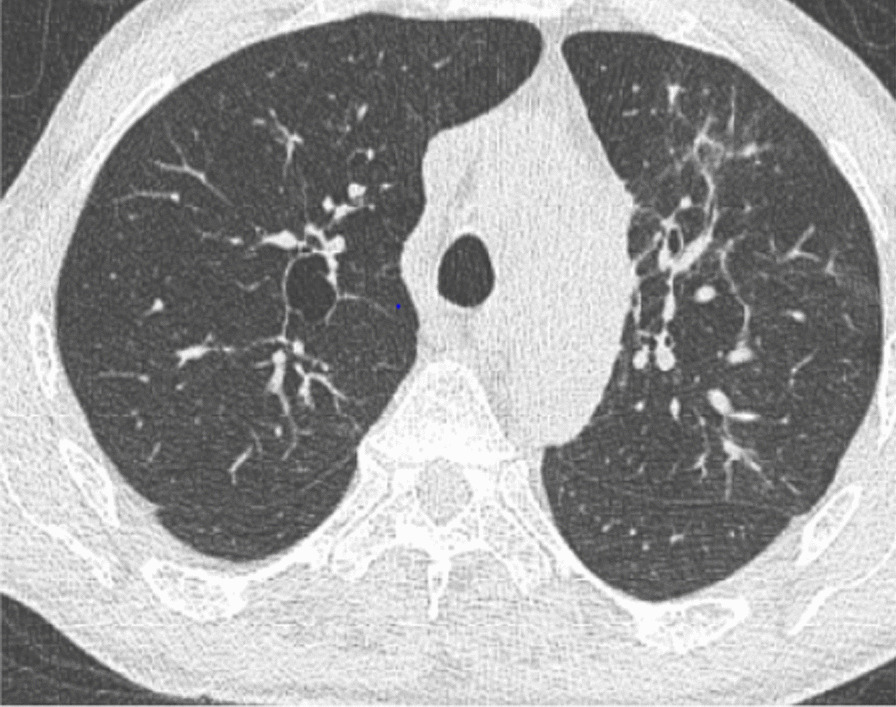
Thoracic CT scan (performed after the second bolus of cyclophosphamide) showed radiological improvement of excavated nodules with the disappearance of condensation

**Fig. 7:**
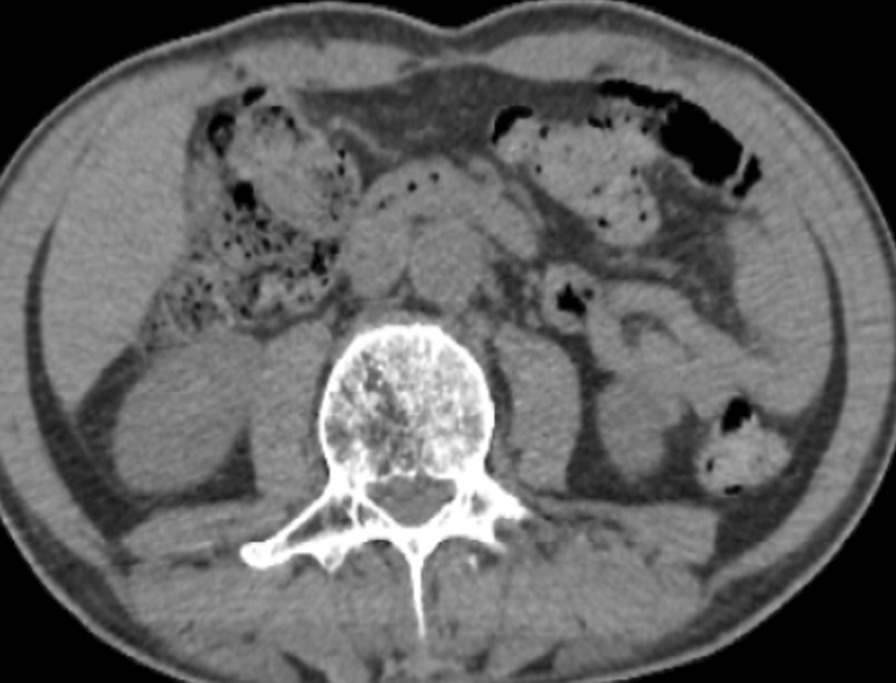
Spontaneous contrast abdominal CT scan (performed after the second bolus of cyclophosphamide) shows clear regression of periaortic tissue infiltrate

The 66-year-old Moroccan male patient had a cumulative smoking index of 30 pack-years. The patient stopped smoking 5 years earlier, had no alcoholism, and had no personal or family history or professional or environmental exposure for bilateral jugular thrombosis. He was put on antivitamin K for 1 month and still developed thoracic pains with productive coughing bringing up hemoptoic sputum, lumbago, dysphonia, epistaxis, and deafness of the left ear of acute installation. At admission, the patient was respiratorily and hemodynamically stable. The general examination on admission revealed a conscious patient with good orientation in time and space, with a heart rate of 63 beats per minute, blood pressure of 120/60 mm Hg, respiratory rate of 16 breaths per minute, and temperature of 37 °C. On neurological examination, mobility, sensitivity, and osteotendinous reflexes were preserved. Examination of the cranial nerves was without anomalies. The rest of the physical examination was unremarkable except for red eyes and some leg purpuric lesions (Table 1). There were no peripheral signs of heart failure and no signs of peritoneal irritation on percussion, and chest X-ray showed several excavated opacities (Fig. 1). The thoracoabdominopelvic computed tomography scan revealed multiple thromboses, particularly in the jugular veins (Fig. 2), extended to the brachiocephalic trunk and the superior vena cava, and in the left common femoral vein, a left intraventricular thrombus. The sinus scan revealed chronic maxillary sinusitis with episcleritis perforation of its medial wall (Fig. 3), excavated bilateral nodular pulmonary lesions (Fig. 4) associated with a periaortic adenopathy flow, and a fly sheathing the aorta suggestive of retroperitoneal fibrosis (Fig. 5).The routine blood and urine analysis detected anemia. Renal and hepatic function were normal, and hepatitis B and C and human immunodeficiency virus (HIV) serologies were negative. There was an increase of the acute phase reactant C-reactive protein (CRP) 188 mg/L (normal value ≤ 6 mg/L), an increase of 24-hour proteinuria to 1.4 g/L (normal value 0.3 g/L), microscopic hematuria detected on cytobacteriological examination of urine, urinary sediment positive for blood and albumin, acid–alcohol resistant bacillus in sputum negative with GeneXpert analysis, antiphospholipid antibodies negative, and anticytoplasmic antibodies of neutrophilic polynuclear antibodies ANCA-PR3 positive to 63 UI/mL. Ear, nose, and throat examination showed a highly inflamed nasal mucosa, suggesting bacterial infection. The patient received antibiotic treatment, the examination also showed subglottic stenosis causing dysphonia. Ophthalmologic examination was indicative of bilateral episcleritis. Transthoracic ultrasound showed ischemic cardiomyopathy, which was complicated by a left intraventricular thrombus and a circumferential effusion with fibrin deposits. Other complementary tests, such as PET scan or biopsy (pulmonary, nasal, renal, retroperitoneal fibrosis), did not lead to suspicion of neoplastic pathology or therapeutic urgency. Based on these results, we diagnosed GPA with cardiovascular, pulmonary, renal, and nasal involvement associated with retroperitoneal fibrosis (Tables 2, 3). In addition to the anticoagulant [warfarin 2 mg with international normalized ratio (INR) of 2], the patient was treated with immunosuppressive therapy [bolus of methylprednisolone 15 mg/kg/day 3 days in a row, bolus of cyclophosphamide Endoxan 500 mg/m^2^ with Uromitexan (mesna) 500 mg/ m^2^), and oral corticosteroids (1 m/kg)], (Tables 4, 5), with clinical improvement including the disappearance of dyspnea and hemoptysis, radiological improvement including regression of excavated nodules (Fig. 6), decrease in the size of the periaortic infiltrate (Fig. 7), and a slight improvement in renal function (24-hour proteinuria of control at 0.7 mg/L) and ANCA levels (ANCA of control at 53 UI/mL) (Table 4). The follow-up was complicated by several relapses, hospitalization, and an increase of corticosteroid therapy

**Table 1:** The chronology of clinical signs

Time	Clinical presentation
T0	Bilateral jugular thrombosis
T1 1 month	Chest pain with productive cough bringing up hemoptoid sputum
T2 1 month and 1 week	Lumbago, dysphonia, epistaxis, deafness of the left ear
T3 1 month and 10 days	Red eyes and some purpuric lesions on the legs

**Table 2:** The diagnostic criteria in our patient

Diagnostic criteria	Arguments
Clinical	Lung: chest pain with hemoptoic sputum, bilateral nodular excavated lung lesions Kidney: microscopic hematuria, positive urine sediment, 24-hour proteinuria ENT: dysphonia, epistaxis, left ear deafness, subglottic stenosis on nasofibroscopy, perforation of the medial sinus wall Ophthalmic: red eyes, bilateral episcleritis Cardiovascular involvement: multiple thrombosis, circumferential pericardial effusion Lumbar: lumbago, retroperitoneal fibrosis
Biology	Anticytoplasmic neutrophil antibodies ANCA-PR3 positive at 63 IU/mL
Histology	Biopsies not done because of the therapeutic emergency and the impossibility of stopping the anticoagulant treatment

*ENT*: Eyes, Nose, Throat; *ANCA-PR3*: anti-neutrophil cytoplasmic antibodies proteinase 3

**Table 3:** Diagnostic criteria

Diagnostic criteria granulomatosis with polyangiitis: should show two or more criteria
Nasal or oral inflammation
Abnormal thoracic X-ray
Active urinary sediment
Granulomatous inflammation in the biopsy

**Table 4:** Clinical course and follow-up visits

Time	Treatment administered	Clinical, biological, and radiological evolution
T0	A bolus of methylprednisolone 3 days in a row	Disappearance of purpuric lesions
T1 day 4	A first bolus of Endoxan + 60 mg prednisone	Disappearance of chest pain Cessation of hemoptysis and epistaxis
T2 2 weeks	Second bolus of Endoxan + 60 mg prednisone	Regression of pulmonary nodules and periaortic infiltration
T3 2 weeks	3rd bolus of Endoxan + 60 mg prednisone	Decrease in cANCA Decrease in 24-hour proteinuria
T4 3 weeks	Fourth bolus of Endoxan + 60 mg prednisone	Regression of jugular thrombosis
T5 3 weeks	Fifth bolus of Endoxan + 40 mg prednisone	Clinical, biological, radiological stability
T6 3 weeks	Sixth bolus of Endoxan + 40 mg prednisone	Clinical, biological, radiological stability
T7 1 week from the end of sixth bolus	Azathioprine 50 mg + 30 mg prednisone	Relapse leading to increase in corticotherapy

**Table 5:** Bolus of Endoxan and monitoring

Hour 0	Hour 1	Hour 2	Hour 3	Hour 4	Hour 5
500 saline serum (SS 0.9%)	Endoxan 1 g/500 cc of glycolic serum (GS 5%) 1/3 solution mesna (600 mg/ 100 cc GS 5%)	500 cc of SS 0.9%	Furosemide 40 mg intravenous	1/3 solution mesna (600 mg/100 cc GS 5%) Drinking water	1/3 solution mesna (600 mg/100 cc GS 5%) Drinking water

Monitoring	Hour 0	Hour 1	Hour 2	Hour 3	Hour 5	Hour 6	Hour 7
Blood pressure	120/60 mmHg	120/60 mmHg	130/70 mmHg	130/70 mmHg	120/60 mmHg	120/60 mmHg	120/60 mmHg
Respiratory frequency	16 breaths per minute	16 breaths per minute	17 breaths per minute	16 breaths per minute	16 breaths per minute	16 breaths per minute	16 breaths per minute
Pulse	63 beats per minute	70 beats per minute	73 beats per minute	75 beats per minute	70 beats per minute	65 beats per minute	65 beats per minute
Temperature	37 °C	37 °C	37 °C	37 °C	37 °C	37 °C	37 °C

## Discussion

Granulomatosis with polyangiitis (Wegener's) is a necrotizing vasculitis combining inflammation of the vascular wall with peri- and extravascular granulomatosis [2]. Despite the discovery of anti-PR3 ANCAs that are highly specific for GPA, the pathogenesis of this disease is still poorly understood; however, neutrophils are the key players because they produce the autoantigen, proteinase 3, and their regulation is altered in patients [2]. Clinically, it is characterized in its full form by ENT signs and lung and kidney involvement. Other systemic manifestations may occur, and the risk of VTE is high [7]. Chest X-rays and CT scans show nodules, single or bilateral, single or multiple, excavated in half of the cases. Single or bilateral pulmonary infiltrates may also appear. Granulomatous pseudotumoral lung masses are possible [2, 6] (Table 1).

The presence of thrombosis in GPA is frequent. Analysis of a randomized controlled trial Wegener's Granulomatosis Etanercept Trial (WGET), which included patients with GPA, showed a VTE incidence of 7.0 per 100 person-years [4]. An increased likelihood of VTE was reported in a population-based incident AAV cohort, which was driven by a significantly increased risk of developing deep venous thrombosis (DVT) [7]. The analysis of a large cohort of patients with eosinophilic granulomatosis with polyangiitis (EGPA), GPA, and MPA demonstrated occurrence of VTEs in 8.2%, 8.0%, and 7.8% of patients, respectively [8]. In a prospective study, Merkel *et al.* [4] found an increased incidence of 7.0/100 person-years of VTE in Wegener granulomatosis (WG) patients. For comparison, in healthy Swedish men, the incidence is 0.3/100 person-years [9]. The cause of this increased incidence of VTE in WG patients cannot be derived from the study of Merkel *et al.* More recently, an analysis of data derived from several trials conducted by the European Vasculitis Society showed VTE occurrence in 41 (9.8%) of 417 patients with GPA or MPA [10]. Hansson *et al.* found an increased incidence of VTE just before and after the diagnosis of AAV of 1.8/100 person-years, compared with 0.3 in a healthy population of the same age [9]. The cause of the increased risk of VTE in AAV patients, especially when the disease is active, is unknown. Changes in endothelial function and hypercoagulability, especially during active disease, could explain this risk of VTE. Both cytokines and ischemia are known to cause endothelial damage [11]. Circulating ANCAs may also cause endothelial damage [12]. Increased platelet aggregation [11] and decreased fibrinolytic capacity [13] during active disease also appeared to cause thrombosis in AAV patients.

Retroperitoneal fibrosis (RPF) is very rare in granulomatosis with polyangiitis. The incidence of RPF is 0.1 per 100,000 population/year, with a prevalence of 1.4 per 100,000 population. Seventy percent of cases are idiopathic [14]. The remaining cases may be secondary to infections, abdominal surgery, medication, or malignant tumors. Recently, idiopathic retroperitoneal fibrosis was reported among the manifestations of immunoglobulin (Ig)G4 disease. This disease is responsible for only a portion of idiopathic retroperitoneal fibrosis cases (50%) [15, 16]. Imaging plays an important role in the diagnosis of RPF, in the distinction between benign and malignant forms, and finally in the monitoring of the evolution under treatment [14]. However, if diagnostic dilemmas exist and an underlying malignancy is suspected, or if there is no response to initial treatment, biopsy must be performed to confirm the diagnosis.

The association of retroperitoneal fibrosis with c-ANCA or p-ANCA positive systemic vasculitis is described in the literature, for granulomatosis with polyangiitis as well as in other vasculitides such as Churg–Strauss syndrome [17]. Revilla *et al.* reported a case of a 74-year-old man with a past infrarenal abdominal aortic aneurysm. Cavitating pulmonary nodules and retroperitoneal fibrosis with periaortic alterations were detected on computed tomography. Laboratory investigations revealed that the patient was positive for cytoplasmic antineutrophil cytoplasmic antibodies (c-ANCA), and necrotizing granulomas were observed on the lung lesion biopsies retroperitoneal tissue. The patient was diagnosed with GPA, and immunosuppressive therapy was prescribed with glucocorticoids 1 mg/kg for 3 days and methotrexate 20 mg per week, which led to a clinical and radiological improvement [2]. Izzedine *et al.* reported the case of a 51-year-old man with abdominal pain, urinary symptoms, and a constitutional syndrome. Imaging revealed retroperitoneal fibrosis. Necrotizing granulomas, giant cells, and a lympho-epithelioid cellular infiltrate were observed on the retroperitoneal tissue biopsy. The patient responded poorly to antituberculous treatment. Finally, a renal biopsy was performed, which revealed pauci-immune rapidly progressive glomerulonephritis with necrotizing vasculitis. Alveolar opacities compatible with alveolar hemorrhages were observed on thoracic CT, and a diagnosis of GPA was made. The patient showed significant improvement after treatment with immunosuppressive agents and corticosteroids [18].

These observations show that, if the clinical presentation is suggestive of vasculitis, the discovery of retroperitoneal fibrosis should not lead to further investigations and will be considered secondary to GPA. Immunosuppressive treatment must be started quickly given the rapid evolution of the disease and the functional and vital prognosis involved. Our observation confirms this notion. The diagnosis of RPF has been attributed to GPA; in this sense, the complementary examinations (PET scan and biopsy) in search of neoplasia or other etiology have not been realized. Our patient responded favorably to immunosuppressive therapy and corticosteroids. The similarly responding retroperitoneal lesion may suggest that this is an unusual presentation in GPA.

## Conclusion

Granulomatosis with polyangiitis is a systemic, multiorgan disease with an increased risk of VTE; however, the association with retroperitoneal fibrosis remains rare, and systematic biopsy does not seem justified in the absence of clinical and/or paraclinical guidance.

## Data Availability

Not applicable.

## Abbreviations

GPA: Granulomatosis with polyangiitis
ENT: Ears, nose, throat
VTE: Venous thromboembolism
c-ANCA: Cytoplasmic antineutrophilic antibodies
PE: Pulmonary embolism
DVT: Deep venous thrombosis
AAV: Cytoplasmic antineutrophilic antibodies-associated vasculitis
HR: Hazard ratio
CI: Confidence interval
CRP: C-reactive protein
WGET: Wegener’s granulomatosis etanercept trial
EGPA: Eosinophilic granulomatosis with polyangiitis
MPA: Microscopic polyangiitis
WG: Wegener granulomatosis
RPF: Retroperitoneal fibrosis
CT-scan: Computed tomography scan
